# Several factors predict the achievement of the patient acceptable symptom state and minimal clinically important difference for patient‐reported outcome measures following anterior cruciate ligament reconstruction: A systematic review

**DOI:** 10.1002/ksa.12460

**Published:** 2024-09-09

**Authors:** Bryan Sun, Prushoth Vivekanantha, Hassaan Abdel Khalik, Darren de SA

**Affiliations:** ^1^ Michael G. DeGroote School of Medicine McMaster University Hamilton Ontario Canada; ^2^ Division of Orthopaedic Surgery, Department of Surgery McMaster University Hamilton Ontario Canada

**Keywords:** anterior cruciate ligament, MCID, MIC, PASS, PROM

## Abstract

**Purpose:**

To summarize the predictors of the patient acceptable symptom state (PASS), minimal clinically important difference (MCID) and minimal important change (MIC) for patient‐reported outcome measures (PROMs) following anterior cruciate ligament reconstruction (ACLR).

**Methods:**

MEDLINE, PubMed and Embase were searched from inception to 5 January 2024. The authors adhered to PRISMA/R‐AMSTAR guidelines, and the Cochrane Handbook for Systematic Reviews of Interventions. Data on statistical associations between predictive factors and PROMs were extracted. Inverse odds ratios (ORs) and confidence intervals (reverse group comparison) were calculated when appropriate to ensure comparative consistency.

**Results:**

Thirteen studies comprising 21,235 patients (48.1% female) were included (mean age 29.3 years). Eight studies comprising 3857 patients identified predictors of PASS, including lateral extra‐articular tenodesis (LET) (OR = 11.08, *p* = 0.01), hamstring tendon (HT) autografts (OR range: 2.02–2.63, *p* ≤ 0.011), age over 30 (OR range: 1.37–2.28, *p* ≤ 0.02), male sex (OR range: 1.03–1.32, *p* ≤ 0.01) and higher pre‐operative PROMs (OR range: 1.04–1.21). Eight studies comprising 18,069 patients identified negative predictors of MCID or MIC, including female sex (OR = 0.93, *p* = 0.034), absence of HT autografts (OR = 0.70, *p* < 0.0001), higher pre‐operative PROMs (OR = 0.76‐0.84, *p* ≤ 0.01), meniscectomy (OR = 0.67, *p* = 0.014) and collision sports (OR = 0.02–0.60, *p* ≤ 0.05).

**Conclusion:**

Higher pre‐operative PROMs, age over 30, male sex, LETs and HT autografts predicted PASS achievement. Lower pre‐operative PROMs, male sex, non‐collision sports, and lack of meniscectomies predicted MCID/MIC achievement. This review provides a comprehensive understanding of the predictors of clinically significant post‐ACLR outcomes, thus improving clinical decision‐making and the management of patient expectations.

**Level of Evidence:**

Level IV.

**Abbreviations**
ACLR: anterior cruciate ligament reconstruction
BPTB: bone‐patellar tendon‐bone
FOHIRL: fixed‐object high‐impact rotation landing
HT: hamstring tendon
IKDC: International Knee Documentation Committee
KOOS: Knee Injury and Osteoarthritis Outcome Score
MINORS: Methodological Index for Non‐randomized Studies
ML: machine learning
n.s: not significant
PRISMA: Preferred Reporting Items for Systematic Reviews and Meta‐analyses
PROM: patient‐reported outcome measure
RCT: randomized controlled trial
R‐AMSTAR: revised assessment of multiple systematic reviews
SD: standard deviation

## INTRODUCTION

Patient‐reported outcome measures (PROMs) are increasingly being used in orthopaedic literature in order to outline the subjective effects of an intervention [1, 46, 60, 61]. While statistical significance may be achieved between pre‐operative and post‐operative PROM scores or when comparing scores across two different interventions, this difference in effect may not be clinically perceived by patients [18, 50]. Two of the most common thresholds used to determine the clinical relevance of changes in PROMs include the patient acceptable symptom state (PASS), minimal clinically important difference (MCID) and minimal important change (MIC) [5, 18, 50]. The MCID defines the minimum difference in PROM scores that is perceived by patients as beneficial. The MIC refers to the same concept but specifically applies to within‐group differences before and after an intervention. However, while the MCID/MIC represents a threshold of significant change, it does not provide information regarding whether patients ultimately attained their self‐defined standards of satisfaction. In other words, patients may experience clinically significant changes, yet remain unsatisfied with their outcome. Moreover, patients may already be satisfied with their pre‐operative condition, meaning that undergoing surgical interventions, even if they provide a clinically significant benefit, is unnecessary. To help address this deficit, researchers may also utilize the PASS, which is defined as the minimum score for a PROM that meets their individual definition of satisfaction and willingness to undergo the intervention again [18, 56]. Both the MCID and PASS may be calculated by both distribution‐based and anchor‐based methods which are well‐established in the literature [24].

There have been several studies that have attempted to predict the achievement of PASS and MCID across a variety of different orthopaedic subspecialties, including hip arthroscopy, arthroplasty, and spine surgery. Traditional linear and logistic regression models are commonly used for these analyses; however, machine learning (ML) models such as neural networks have also gained popularity due to various purported benefits (e.g., improved modelling of non‐linear relationships) [21, 27, 33]. Anterior cruciate ligament reconstruction (ACLR) is one of the most common procedures performed worldwide; however, the role of operative versus non‐operative intervention remains controversial. This is likely related to the paucity of literature assessing how patient demographics (e.g., sex, age and comorbidities) and operative management strategies (e.g., graft choice, concomitant procedures and rehabilitation protocol) influence the likelihood of achieving clinically meaningful improvements [29]. Thus, identifying the demographic and treatment‐related factors that contribute to achieving the PASS or MCID after ACLR is critical as it would enable clinicians to effectively identify which patients and which set of individualized treatment decisions are most likely to result in clinically significant benefits [23, 29]. Moreover, a focus on clinical significance is especially relevant in sports medicine procedures like ACLR, as patients tend to have high functional demands and expectations [29]. Therefore, this systematic review aims to summarize the literature on demographic and surgical factors that contribute to achieving clinically significant outcomes after ACLR.

## MATERIALS AND METHODS

The Preferred Reporting Items for Systematic Reviews and Meta‐Analyses (PRISMA) and Revised Assessment of Multiple Systematic Reviews (R‐AMSTAR) guidelines for coordinating and reporting systematic reviews were followed during the development of this research [25, 34].

### Search criteria

Three online databases (PubMed, MEDLINE and Embase) were searched on 5 January 2024, to identify literature outlining statistical associations between pre‐operative, intraoperative or post‐operative factors and achievement of the PASS, MCID, and related factors of different PROMs in ACLR. The PASS was defined as the minimum score for a PROM that meets a patient's individual definition of satisfaction and willingness to undergo the intervention again [18]. The MCID was defined as the minimum difference in PROM scores that is perceived by patients as beneficial [18]. The term MIC was defined in the same way as the MCID, given that both terms have been defined heterogeneously in the published literature [23]. Comprehensive search terms, including ‘anterior cruciate ligament’, ‘minimal clinically important difference’, ‘minimal important change’ and ‘patient acceptable symptom state’ were utilized (Supporting Information S1: Table 1).

Studies were selected for inclusion if they met the following criteria: (1) studies with patients undergoing primary ACLR (including pediatric and multi‐ligament injury patients), (2) studies using traditional regression or ML models to outline statistical associations between pre‐operative, intraoperative or post‐operative factors and achievement of the PASS, MCID, or related measures (e.g., minimal important change or MIC) for Knee Osteoarthritis and Outcome Score (KOOS) subscales, Lysholm and International Knee Documentation Committee (IKDC) [19, 49, 59], (3) human studies, and (4) studies published in the English language. Exclusion criteria included (1) patients undergoing ACL repair, (2) level of evidence V, (3) textbook chapters, systematic reviews or meta‐analyses, (4) conference abstracts and (5) biomechanical, cadaveric or animal studies. The reference sections of included studies were also assessed for possible eligibility.

### Screening

Title and abstract screening were independently and blindly conducted by two authors (BS and PV), with conflicts resolved through consensus or consultation with a more senior author (DDS) if no consensus was reached. During the full‐text screening stage, studies were independently screened by the initial two authors, and disagreements were resolved in a similar manner.

### Assessment of agreement

The inter‐reviewer agreement was evaluated using the kappa (*κ*) statistic for screening. A priori classification was determined using the following criteria: *κ* of 0.91–0.99 was considered to be almost perfect agreement; *κ* of 0.71–0.90 was considered to be considerable agreement; *κ* of 0.61–0.70 was considered to be high agreement; *κ* of 0.41–0.60 was considered to be moderate agreement; *κ* of 0.21–0.40 was considered to be fair agreement, and a *κ* value of 0.20 or less was considered to be no agreement [37].

### Quality assessment

Quality assessment of included studies was assessed using the Methodological Index for Non‐Randomized Studies (MINORS) criteria [55]. Based on the MINORS criteria, non‐comparative and comparative studies could get a maximum score of 16 and 24, respectively [55]. For non‐comparative studies, classification was a priori based on a previous systematic review: scores of 0–4 indicated very low‐quality evidence; 5–7 indicated low‐quality evidence; 8–12 indicated fair quality; ≥13 indicated high‐quality evidence. Comparative studies were classified as follows: scores of 0–6 indicated very low‐quality evidence; 7–10 indicated low‐quality evidence; 11–15 indicated fair‐quality evidence; 16–20 indicated good‐quality evidence and ≥20 indicated high quality [6].

### Data abstraction and reporting

Data were extracted in an electronic spreadsheet designed *a* priori (Google Sheets, Google LLC). Demographic data such as number of patients, follow‐up time, sex and mean age were recorded. Outcomes included definitions of clinically significant outcomes, MCID and PASS values for KOOS subscales, Lysholm and IKDC scores, associated calculation methods (e.g., anchor‐based and distribution‐based methods) and statistical associations between pre‐operative, intraoperative or post‐operative factors and achievement of PASS or MCID. The statistical association was recorded in the form of correlation coefficients, odds ratios (ORs), receiver operator curve (ROC) analyses, or by importance rank (ML). The significance of statistical associations was recorded in the form of *p* values, with values below 0.05 indicating significance.

Inverse ORs (reverse group comparison) and their associated confidence intervals were calculated using Google Sheets (Google LLC) to ensure comparative consistency when necessary. For example, if the majority of studies reported ORs as male sex (numerator) versus female sex (denominator), the inverse ORs would be calculated for the remaining studies which reported ORs as female sex (numerator) versus male sex (denominator). Confidence intervals, means, ranges, percentages and standard deviations (SDs) were also calculated using Google Sheets (Google LLC). Results were presented in a descriptive summary format.

## RESULTS

### Literature search

**Figure 1:**
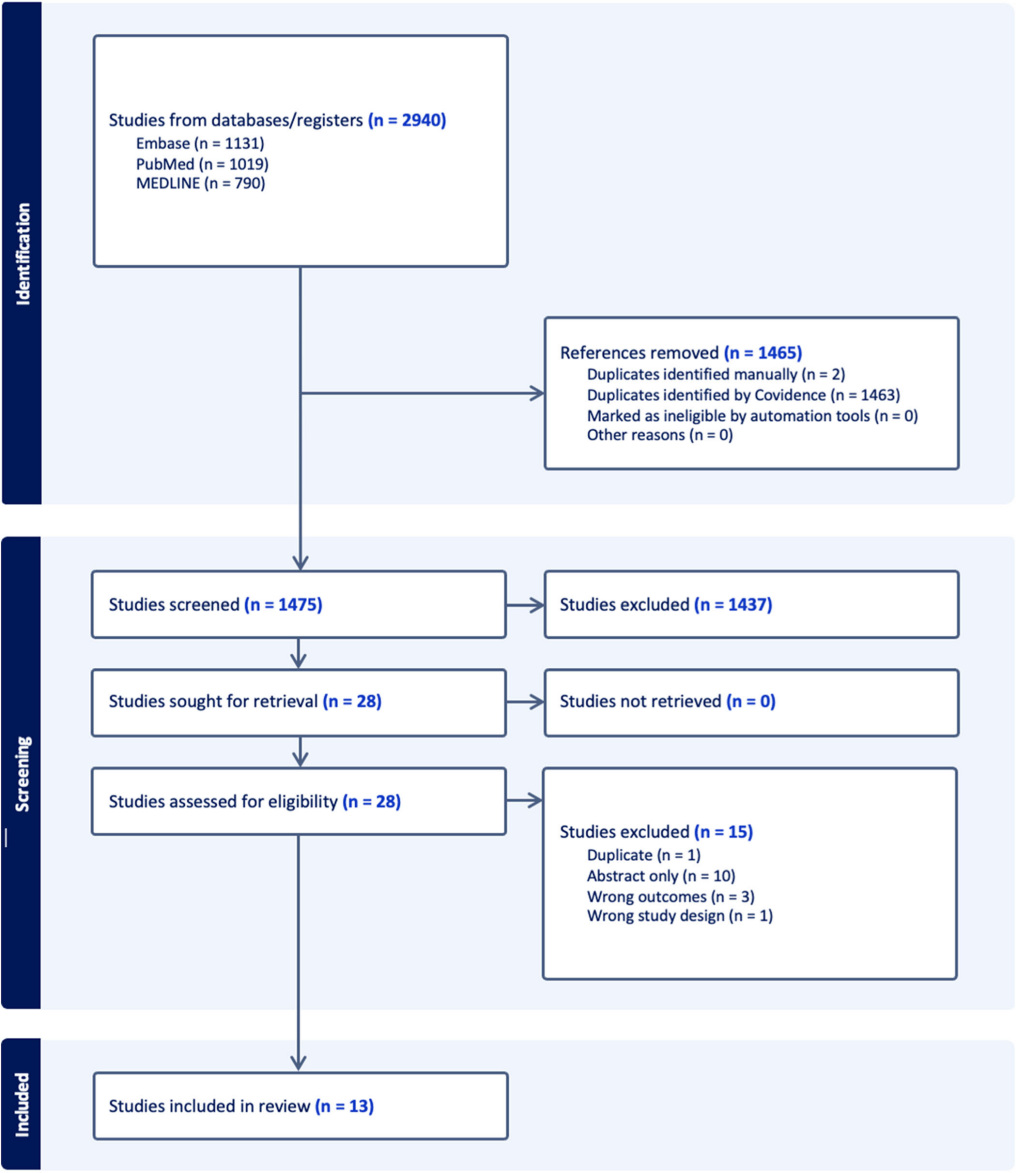
Preferred Reporting Items for Systematic Reviews and Meta‐analyses flow diagram representing a systematic review evaluating factors predicting the achievement of the patient acceptable symptom state, minimal clinically important difference, or minimal important change for patient‐reported outcome measures after anterior cruciate ligament reconstruction.

The initial search of the databases yielded 2940 studies, with 1465 being automatically filtered out as duplicates. Following title and abstract screening, 1,437 studies were deemed irrelevant, leaving 38 studies for full‐text screening. Ultimately, 13 full‐text articles satisfied the inclusion and exclusion criteria [3, 7, 12, 13, 16, 17, 23, 29, 36, 40, 42, 47, 64] (Figure 1). There was strong agreement during both the title and abstract stage (*k* = 0.802, 95% confidence interval [CI] = 0.713–0.891) and during the full‐text stage (*k* = 0.833, 95% CI = 0.652–1.000).

### Study quality

Thirteen studies in this review included three case series (level of evidence IV) [36, 42, 47], three retrospective cohorts (Level III evidence) [3, 12, 23], six case–controls (Level III evidence) [7, 13, 17, 29, 40, 64], and one prospective cohort (Level II evidence) [16]. The mean MINORS score was 10.1/16 and studies were considered fair quality (Table 1).

**Table 1:** Demographics.

Author (year)	Study design (level of evidence)	Number of patients/knees	Follow‐up time ± SD (months)	Female (%)	Mean age ± SD (years)	Loss to follow‐up (%)	MINORS (/16)
Beletsky (2021)	Retrospective cohort (III)	144/144	6, 12, 24	90 (62.5)	30.9 (12.8)	NR	10
Cristiani (2020)	Case–control (III)	2335/2335	Minimum 24	1153 (49.4)	29.7 (10.9)	48.7	11
Forlenza (2020)	Retrospective cohort study (III)	253/253	12	124 (49.1)	36.2 (14.7)	NR	11
Forsythe (2021)	Case–control (III)	379/379	Minimum 12	178 (47.0)	34.3 (14.1)	38	11
Senorski (2019)	Prospective study (II)	147/147	196.8 (15.6)	52 (35.4)	27.3 (8.3)	NR	10
Senorski (2018)	Case–control (III)	343/343	12	176 (51.3)	28.1 (10.6)	8.1	9
Kaarre (2023)	Retrospective cohort (III)	16,131/16,131	12	7857 (48.7)	28.9 (10.7)	NR	9
Kunze (2021)	Case–control study (III)	442/442	Minimum 24	211 (47.7)	Median (IQR): 29.0 (21.0–40.3)	NR	9
Maheshwer (2022)	Case series (IV)	59/59	24	34 (57.6)	Median (IQR): 16.0 (14.5–17.0)	NR	10
Nwachukwu (2017)	Case–control (III)	231/231	24	112 (48.5)	26.7 (12.5)	NR	11
Nwachukwu (2021)	Retrospective review (IV)	142/142	24, 92.9 (8.7)	71 (50.0)	27.2 (13.0)	NR	10
Puzzitiello (2023)	Retrospective review (IV)	197/197	74.5 (25.2)	102 (51.8)	48.5 (5.6)	NR	9
Ye (2022)	Case–control (III)	432/432	72.0 (37.2)	112 (25.9)	26.8 (8.4)	1.9	11

Abbreviations: IQR, interquartile range; MINORS, methodological index for non‐randomized studies; SD, standard deviation.

### Study characteristics

Thirteen studies were included in this review, comprising a total of 21,235 patients [3, 7, 12, 13, 16, 17, 23, 29, 36, 40, 42, 47, 64]. Eight studies reported minimum follow‐up periods ranging from 4 to 24 months [7, 12, 13, 17, 23, 29, 36, 40], while four studies had a mean final follow‐up of 109.1 months [16, 42, 47, 64]. One other study reported follow‐up times at 6, 12 and 24 months [3]. All studies reported on patient sex, with females comprising 48.1% of 21,235 patients. Eleven studies comprising 20,734 patients reported on patient age at index surgery, with the weighted mean being 29.3 years (range of means: 26.7–48.5) [3, 7, 12, 13, 16, 17, 23, 40, 42, 47, 64]. Loss to follow‐up was reported in four studies ranging from 1.9% to 48.7% [7, 13, 17, 64].

Eight studies comprising 3857 patients reported on factors predicting PASS [3, 7, 12, 13, 16, 17, 36, 47]. Six of these studies used PASS thresholds derived from anchor‐based methods [3, 7, 12, 17, 36, 47], one used anchor and distribution‐based methods [13], and one was unclear [53]. Eight studies comprising 18,069 patients reported on factors predicting MCID or MIC [12, 13, 23, 29, 36, 40, 42, 64]. Five of these studies used MCID/MIC thresholds derived from distribution‐based methods [29, 36, 40, 42, 64], one used anchor‐based methods [23], one used both [13] and one was unclear [12]. Three studies comprising 691 patients reported on both PASS and MCID. In terms of statistical inferencing, six studies comprising 3312 patients used multivariate analysis [3, 7, 12, 13, 36, 42], five studies comprising 17,049 patients used multivariable analysis [16, 17, 23, 40, 47], and two studies comprising 874 patients used ML models [29, 64] (Table 1).

### Patient acceptable symptom state

#### Demographic factors

Four studies comprising 2929 patients reported on demographic factors predicting the achievement of the PASS [3, 7, 12, 47]. One study reported that the male sex increased the likelihood of achieving PASS for KOOS QoL (OR = 1.03, *p* < 0.001), while another study reported that the male gender similarly increased the likelihood of achieving PASS for KOOS Pain (OR = 1.32, *p* = 0.01) and KOOS Sports and Recreation (S/R) (OR = 1.39, *p* = 0.003) [3, 7]. One study reported that baseline diabetes mellitus decreased the likelihood of achieving PASS for KOOS ADL (OR = 0.05, *p* = 0.014), while another study reported that higher BMI increased the likelihood of not achieving PASS for IKDC (OR = 1.12, *p* = 0.013) [3, 47]. One study reported that worker's compensation status decreased the likelihood of achieving PASS for IKDC (OR = 0.25, *p* = 0.033) and KOOS ADL (OR = 0.28, *p* = 0.041), while participation in pre‐operative exercises increased the likelihood of achieving PASS for IKDC (OR = 4.74, *p* = 0.003) and KOOS ADL (OR = 2.95, *p* = 0.038) [3]. One study reported that patient age greater than or equal to 30 years increased the likelihood of achieving PASS for all KOOS subscales (OR range: 1.37–2.28, *p* ≤ 0.02) [7]. A final study found that pre‐operative opioid use decreased the likelihood of achieving PASS for all KOOS subscales (OR range: 0.23–0.56, *p* ≤ 0.02) [12] (Table 2).

**Table 2:** Significant predictors of PASS.

Author (year)	Analysis type	PROM	PASS value (SD)	PASS calculation	Achieving or non‐achieving	Significant predictive factors and statistical associations (95% CI)
Beletsky (2021)	Multi‐ variate	IKDC	75 (0.7)	Anchor	Achieving	Pre‐operative exercise: OR = 4.74 (1.69–13.34), *p* = 0.003 Worker's compensation status: OR = 0.25 (0.07–0.88), *p* = 0.033 Higher pre‐operative IKDC: OR = 1.07 (1.04–1.11), *p* < 0.001 Higher pre‐operative Lysholm score: OR = 1.05 (1.02–1.08), *p* < 0.001
		KOOS JR	76.3 (0.9)			Anteromedial technique: OR = 1.05 (1.01–1.10), *p* < 0.001
		KOOS PS	18.6 (0.8)			Iliotibial band tenodesis: OR = 11.08 (1.76–69.77), p = 0.010 Anteromedial technique: OR = 1.08 (1.02–1.13), *p* < 0.001
		KOOS ADL	92.3 (0.8)			Pre‐operative exercise: OR = 2.95 (1.06–8.19), *p* = 0.038 Diabetes mellitus: OR = 0.05 (0.01–0.54), *p* = 0.014 Worker's compensation status: OR = 0.28 (0.08–0.95), *p* = 0.041 Higher pre‐operative KOOS ADL: OR = 1.04 (1.01–1.07), *p* = 0.005
		KOOS S/R	70.0 (0.8)			Higher pre‐operative KOOS S/R: OR = 1.21 (1.12–1.30), *p* < 0.001
		KOOS QoL	50.0 (0.8)			Male sex: OR = 1.03 (1.02–1.04), *p* < 0.001
Cristiani (2020)	Multi‐ variate	KOOS pain	88.9	Anchor	Achieving	Age ≥30: OR = 1.54 (1.23–1.92), *p* < 0.001 Female gender: OR = 0.76 (0.62–0.94), *p* = 0.01 [Inverse: Male gender: OR = 1.32 (1.06–1.61), *p* = 0.01] Medial meniscal repair: OR = 0.59 (0.36–0.96), *p* = 0.03 Post‐operative quadriceps strength LSI ≥ 90%: OR = 1.35 (1.08–1.70), *p* = 0.009
		KOOS symptoms	57.1			Age ≥30: OR = 1.68 (1.06–2.67), *p* = 0.02 Post‐operative quadriceps strength LSI ≥ 90%: OR = 1.62 (1.00–2.63), *p* = 0.04
		KOOS ADL	100.0			Age ≥30: OR = 1.37 (1.10–1.71), *p* = 0.004
		KOOS S/R	75.0			Age ≥30: OR = 1.52 (1.20–1.91), *p* < 0.001 Male gender: OR = 1.39 (1.12–1.72), *p* = 0.003 HT autograft vs. BPTB: OR = 2.02 (1.31–3.10), *p* = 0.001 Cartilage injury: OR = 0.73 (0.55–0.97), *p* = 0.03 Post‐operative quadriceps strength LSI ≥ 90%: OR = 1.51 (1.18–1.92), *p* = 0.001 Post‐operative single leg hop test LSI ≥ 90%: OR = 1.40 (1.11–1.77), *p* = 0.004
		KOOS QoL	62.5			Age ≥30: OR = 2.28 (1.78–2.92), p < 0.001 Post‐operative quadriceps strength LSI ≥ 90%: OR = 1.48 (1.15–1.91), *p* = 0.002 Single leg hop test LSI ≥ 90%: OR = 1.28 (1.00–1.63), *p* = 0.04
Forlenza (2020)	Multi‐ variate	KOOS S/R	70.0	Anchor	Non‐achieving	Pre‐operative opioid use: OR = 0.25 (0.09–0.69), *p* = 0.02
		KOOS pain	80.6			Pre‐operative opioid use: OR = 0.31 (0.13–0.77), *p* = 0.02
		KOOS symptoms	78.6			Pre‐operative opioid use: OR = 0.32 (0.14–0.69), p = 0.03
		KOOS QoL	50.0			Pre‐operative opioid use: OR = 0.56 (0.13–2.51), *p* = 0.005
		KOOS ADL	92.3			Pre‐operative opioid use: OR = 0.23 (0.09–0.58), *p* = 0.008
		KOOS JR	76.3			Pre‐operative opioid use: OR = 0.31 (0.11–0.84), *p* = 0.04
Forsythe (2021)	Multi‐ variate	IKDC	75.0	Anchor or distribution	Achieving	1 year: Increased patient symptom duration: OR = 0.56 (0.42–0.92), *p* = 0.03 2 year: Increased patient symptom duration: OR = 0.15 (0.05–0.43), *p* < 0.001
		KOOS pain	80.6			1 year: Increased patient symptom duration: OR = 0.71 (0.50–0.91), *p* = 0.001 2 year: Increased patient symptom duration: OR = 0.41 (0.13–0.72), *p* = 0.007
		KOOS symptoms	78.6			2 year: Increased patient symptom duration: OR = 0.51 (0.14–0.74), *p* = 0.005
		KOOS ADL	92.3			2 year: Increased patient symptom duration: OR = 0.05 (0.00–1.05), *p* = 0.042
Senorski (2019)	Multi‐ variable	IKDC	75.9	NR	Achieving	Concomitant injury: OR = 2.61 (1.10–6.21), *p* = 0.03 Greater pre‐operative anteroposterior translation: OR = 1.87 (1.05–3.35), *p* = 0.034
Senorski (2018)	Multi‐ variable	KOOS pain	88.9	Anchor	Achieving	BPTB vs. HT autograft: OR = 0.44 (0.22–0.90), *p* = 0.026 [Inverse: HT vs. BPTB autograft: OR = 2.27 (1.11–4.55), *p* = 0.026]
		KOOS QoL	62.5			Absence of meniscal injury: OR = 1.62 (1.04–2.54), *p* = 0.035 [Inverse: Presence of meniscal injury: OR = 0.62 (0.39–0.96), *p* = 0.035] BPTB vs. HT autograft: OR = 0.38 (0.18–0.80), *p* = 0.011 [Inverse: HT vs. BPTB autograft: OR = 2.63 (1.25–5.56), *p* = 0.011]
Maheshwer (2022)	Multi‐ variate	KOOS symptoms	75.0	Anchor	Achieving	FOHIRL sport: OR = 0.41 (0.21–0.79), *p* = 0.011
Puzzitiello (2023)	Multi‐ variable	IKDC	66.7	Anchor	Non‐achieving	Higher BMI: OR = 1.12 (1.03–1.23), *p* = 0.013 Lateral compartment cartilage defect: OR = 5.1 (1.87–13.9) *p* = 0.001

Abbreviations: 95% CI, 95% confidence interval; ADLs, activities of daily living; IKDC, International Knee Documentation Committee; JR, joint replacement; KOOS, Knee Injury and Osteoarthritis Outcome Score; NR, not reported; OR, odds ratio; PASS, patient acceptable symptom state; PROM, patient‐reported outcome measure; PS, short form, QoL, quality of life; S/R, sports and recreation; SD, standard deviation.

#### Injury‐related factors

Seven studies comprising 3604 patients reported on injury‐related factors predicting the achievement of the PASS [3, 7, 13, 16, 17, 36, 47]. Two studies found that concomitant cartilage injuries decreased the achievement of PASS for KOOS QoL (OR = 0.62, *p* = 0.035) and KOOS S/R (OR = 0.73, *p* = 0.03). One other study reported concomitant cartilage injury increased the odds of not achieving the PASS for IKDC (OR = 0.51, *p* = 0.001) [7, 17, 47]. In contrast, a fourth study reported that any concomitant injury increased the likelihood of achieving PASS for IKDC (OR = 2.61, *p* = 0.03), although this study's multivariable regression model demonstrated poor goodness‐of‐fit (i.e., the model has limited ability to predict the outcome of interest) [16, 48]. One study reported that higher pre‐operative IKDC and Lysholm levels increased the likelihood of achieving PASS for IKDC (OR = 1.07 and OR = 1.05, respectively, *p* < 0.001), and higher pre‐operative KOOS S/R and ADL increased the likelihood of achieving PASS for KOOS S/R (OR = 1.21, *p* < 0.001) and ADL (OR = 1.04, *p* = 0.005), respectively [3]. One study reported that greater pre‐operative anteroposterior translation increased the likelihood of achieving PASS for IKDC (OR = 1.87, *p* = 0.034), while another study found that participation in fixed‐object high‐impact rotation landing (FOHIRL) sports (i.e., gymnastics) decreased the likelihood of achieving PASS for KOOS Symptoms (OR = 0.41, *p* = 0.011) [16, 36]. Finally, another study reported that increased symptom duration (i.e., time to surgery) decreased the likelihood of achieving PASS for IKDC (OR range: 0.15‐0.56, *p* ≤ 0.03), KOOS Pain (OR range: 0.41–0.71, *p* ≤ 0.007), KOOS Symptoms (OR = 0.51, *p* = 0.005) and KOOS ADL (OR = 0.05, *p* = 0.042) [13] (Table 2).

#### Intraoperative factors

Three studies comprising 2822 patients reported on intra‐operative factors predicting the achievement of the PASS [3, 7, 17]. Two studies reported that ACLR with hamstring tendon (HT) autograft instead of bone‐patellar‐tendon‐bone (BPTB) autograft increased the likelihood of achieving PASS for the KOOS S/R (OR = 2.02, *p* = 0.001), KOOS Pain (OR = 2.27, *p* = 0.026) and KOOS QoL (OR = 2.63, *p* = 0.011) [7, 17]. One study reported that concomitant medial meniscus repairs reduced the likelihood of achieving PASS for KOOS Pain (OR = 0.59, *p* = 0.03) [7]. Finally, one study reported that concomitant iliotibial band tenodesis (LET) increased the likelihood of achieving PASS for KOOS PS (OR = 11.08, *p* = 0.010) and that using an anteromedial tunnel drilling technique increased the likelihood of achieving PASS for KOOS JR (OR = 1.05, *p* < 0.001) and KOOS PS (OR = 1.08, *p* < 0.001) [3] (Table 2).

#### Post‐operative factors

One study comprising 2335 patients reported on post‐operative factors predicting the achievement of the PASS [7]. Post‐operative isokinetic quadriceps strength symmetry, defined as a limb symmetry index (LSI) greater than or equal to 90%, increased the likelihood of achieving PASS for all KOOS subscales (OR range: 1.35–1.62, *p* ≤ 0.04). Post‐operative single leg hop test symmetry, defined in the same manner, also increased the likelihood of achieving PASS for KOOS S/R (OR = 1.40, *p* = 0.004) and KOOS QoL (OR = 1.28, p = 0.04) (Table 2).

### Minimal clinically important difference and minimal important change

#### Demographic factors

Four studies comprising 17,236 patients reported on demographic factors predicting the achievement of the MCID or MIC [23, 29, 40, 64]. Two studies reported that older age decreased likelihood of achieving MCID for IKDC (OR = 0.92, *p* < 0.05) and Lysholm, while one study reported that older age decreased odds of not achieving the MIC for KOOS S/R and QoL composite, henceforth referred to as KOOS composite (OR = 0.91, *p* < 0.0001) [23, 40, 64]. One study found that male sex decreased the likelihood of not achieving the KOOS composite MIC (OR = 0.93, *p* = 0.034) [23]. One ML study reported that BMI over 27.4 decreased the likelihood of achieving the IKDC MCID (the fifth most predictive factor) [29] (Table 3).

**Table 3:** Significant predictors Of MCID/MIC.

Author (year)	Analysis type	MCID or MIC	PROM	PASS value (SD)	PASS calculation	Achieving or non‐achieving	Significant predictive factors and statistical associations (95% CI)
Kaarre (2023)	Multi‐ variable	MIC	KOOS S/R and KOOS QoL Composite Outcome	KOOS S/R: 12.1 KOOS QoL: 18.3	Anchor	Non‐achieving	Older age: OR = 0.91 (0.88–0.94), *p* < 0.0001 Male sex: OR = 0.93 (0.87–0.99), *p* = 0.034 HT vs. BPTB autograft: OR = 0.70 (0.60–0.81), *p* < 0.0001 Concomitant cartilage injury: OR = 1.17 (1.09–1.27), p < 0.0001 Higher pre‐operative KOOS S/R and QoL: OR = 1.34 (1.31–1.36), *p* < 0.0001
Kunze (2021)	ML	MCID	IKDC	9.2	Distribution	Non‐achieving	Previous contralateral knee surgery: most predictive Knee extension loss or recurvatum deformity: 2nd most predictive MCL Grades 1–3: 3rd most predictive Suspensory femoral fixation: 4th most predictive BMI > 27.4: 5th most predictive Pre‐operative IKDC > 62.1: 6th most predictive
Maheshwer (2022)	Multi‐ variate	MCID	IKDC	33.3	Distribution	Achieving	Collision sport: OR = 0.60 (0.375–0.95), *p* = 0.033 FOHIRL sport: OR = 0.53 (0.29–0.97), *p* = 0.046 Meniscectomy: OR = 0.67 (0.49–0.97), *p* = 0.014
			KOOS Sports	45.0			Collision sport: OR = 0.60 (0.37–0.98), *p* = 0.045 Meniscectomy: OR = 0.67 (0.48–0.92), p = 0.017
Nwachukwu (2017)	Multi‐ variable	MCID	IKDC	9.0	Distribution	Achieving	Higher pre‐operative IKDC: OR = 0.76 (0.63–0.86), *p* < 0.0001 Older age: OR = 0.92 (0.85–0.98), *p* < 0.05 Increasing pre‐operative SF‐12 PCS: OR = 1.23 (1.06–1.49), *p* < 0.05 Increasing pre‐operative SF‐12 MCS: OR = 1.27 (1.11–1.52), *p* < 0.05
			Lysholm	10.0			Increasing pre‐operative Lysholm: OR = 0.84 (0.79–0.88), *p* < 0.001 Increasing pre‐operative SF‐12 MCS: OR = 1.08 (1.00–1.16), *p* < 0.05
Nwachukwu (2021)	Multi‐ variate	MCID	IKDC	8.7	Distribution	Achieving	Higher pre‐operative IKDC: OR = 0.83 (0.72–0.96), *p* = 0.01
			Lysholm	10.1			Skiing vs. cutting sport: OR = 43.4 (2.0–927.8), *p* < 0.05 [Inverse: Cutting sport vs. skiing: OR = 0.02 (0.00–0.50), *p* < 0.05]
Ye (2022)	ML	MCID	IKDC	10.4	Distribution	Non‐achieving	Most important predictors: High pre‐operative IKDC, meniscal reinjury after ACLR, short time from injury to surgery, lateral meniscal resection
			Lysholm	10.7			Most important predictors: High pre‐operative Lysholm, older age, short time from injury to surgery, high pre‐operative IKDC

Abbreviations: 95% CI, 95% confidence interval; IKDC, International Knee Documentation Committee; KOOS, Knee Injury and Osteoarthritis Outcome Score; MCID, minimal clinically important difference; OR, odds ratio; PROM, patient‐reported outcome measure; QoL, quality of life; S/R, sports and recreation; SD, standard deviation.

#### Injury‐related factors

Six studies comprising 17,437 patients reported on injury‐related factors predicting the achievement of the MCID [23, 29, 36, 40, 42, 64]. Four studies reported that higher pre‐operative IKDC and Lysholm scores decreased the likelihood of achieving their IKDC (OR range: 0.76–0.83, *p* ≤ 0.01) and Lysholm (OR = 0.84, *p* < 0.001) MCIDs, respectively [29, 40, 42, 64]. On the contrary, one study found that higher Short Form‐12 (SF‐12) physical component scores increased the likelihood of achieving the IKDC MCID (OR = 1.23, *p* < 0.05) and that higher SF‐12 mental component scores increased the likelihood of achieving the IKDC (OR = 1.27, *p* < 0.05) and Lysholm (OR = 1.08, *p* < 0.05) MCIDs [40, 42]. One study found that higher pre‐operative KOOS composite scores increased the likelihood of not achieving its respective MIC (OR = 1.34, *p* < 0.0001) [23]. One study reported that participating in cutting sports instead of skiing decreased the likelihood of achieving the Lysholm MCID (OR = 0.02, *p* < 0.05) [42]. Another study reported that participation in collision sports (i.e., football, lacrosse, ice hockey and wrestling) decreased the likelihood of achieving the IKDC (OR = 0.60, p = 0.033) and KOOS Sports (OR = 0.60, *p* = 0.045) MCIDs, and that participation in FOHIRL sports decreased the likelihood of achieving the IKDC MCID (OR = 0.53, *p* = 0.046) [36]. One ML study reported that previous contralateral knee surgery (most predictive factor), knee extension loss or recurvatum deformity (second most predictive factor), and Grades 1–3 MCL injury (third most predictive factor) all decreased the likelihood of achieving the IKDC MCID [29]. Another ML study reported that a shorter time from injury to surgery also decreased the likelihood of achieving the IKDC MCID [64]. Finally, one study reported that concomitant cartilage injuries increased the likelihood of not achieving the MIC for the KOOS composite (OR = 1.17, *p* < 0.0001) [23] (Table 3).

#### Intraoperative factors

Four studies comprising 17,064 patients reported on intra‐operative factors predicting the achievement of the MIC or MCID [23, 29, 36, 64]. Two studies reported that concomitant meniscectomies decreased the likelihood of achieving the KOOS Sports (OR = 0.67, *p* = 0.017) and IKDC (OR = 0.67, *p* = 0.014) MCIDs, respectively [36, 64]. One ML study found that suspensory fixation decreased the likelihood of achieving the IKDC MCID (fourth most predictive variable). Lastly, one study found that using an HT autograft instead of a BPTB autograft decreased the likelihood of not achieving the KOOS composite MIC (OR = 0.70, *p* < 0.0001) [23, 29] (Table 3).

#### Post‐operative factors

One ML study comprising 432 patients reported on post‐operative factors predicting the achievement of the MCID [64]. The only predictive factor identified was meniscal reinjury following ACLR, which decreased the likelihood of achieving the IKDC MCID (Table 3).

## DISCUSSION

The primary finding of this review was that achievement of the PASS, MCID, and MIC for post‐ACLR IKDC, Lysholm, and KOOS was successfully predicted by various factors. The most important demographic or injury‐related factors were older age (i.e., above 30), male sex, lack of concomitant injuries, and lack of collision/FOHIRL/cutting sports participation. In terms of pre‐operative PROMs, higher values predicted PASS, whereas lower values predicted MCID and MIC. The most significant and commonly reported intraoperative and post‐operative predictive factors were HT autograft use, concomitant LETs, lack of concomitant meniscectomies, and post‐operative LSI >90% for quadriceps strength and single‐leg hop testing. The literature suggests that the ideal patient with regards to PASS and MCID is one who suffers an isolated ACL injury without concomitant cartilage damage, participates in a non‐FOHIRL/cutting sport, and also receives an HT autograft with concomitant LET if indicated as opposed to BPTB autograft. Pre‐operative PROM scores and age were shown to have contradictory results with regard to either PASS or MCID.

Previous studies have reported young age and male sex to be the main demographic factors associated with superior PROMs following ACLR [31, 53]. While this review similarly found that male sex increased the likelihood of achieving clinically meaningful outcomes, the effect of patient age was more complex; older age increased the likelihood of achieving the PASS and MIC yet decreased the likelihood of achieving the MCID. This may be attributable to the heterogeneity in PROMs and age thresholds among the included studies. However, another explanation is that older patients have lower activity and functional standards, meaning they may be more likely to perceive an acceptable symptom state despite small absolute changes in PROMs [56]. Furthermore, there is a growing body of evidence supporting the role of pre‐operative PROMs as predictors of post‐ACLR outcomes [4, 41]. This review is in agreement and found that pre‐operative IKDC, Lysholm, KOOS and SF‐12 predicted the achievement of the PASS for post‐operative PROMs, although contrasting findings were reported for pre‐operative PROMs in relation to the MCID and MIC. Similar to patient age, perhaps patients with higher baseline PROMs are more likely to have acceptable symptoms post‐operatively, while their MCID thresholds would be increased and achieved less frequently, possibly implying that surgery may not have been necessary to achieve PASS.

As the number of ACLRs performed annually continues to increase, it becomes imperative to have evidence‐based management strategies [45]. Optimal surgical timing remains controversial, although many argue against prolonged delays given concerns of subsequent meniscal and chondral injury [65]. However, one included ML study actually found that shorter times to surgery decreased the likelihood of achieving the MCID. While seemingly conflicting, this finding may reflect that surgical timing alone plays a relatively minor role in predicting patient outcomes. Rather, in accordance with the literature, it may be the quality of pre‐operative care or ‘prehabilitation’, which includes optimizing range of motion, mental preparation, resolving post‐injury effusions and so on, that plays the more influential role [8]. In terms of graft selection, using HT instead of BPTB autografts was found to predict the achievement of the PASS and MIC, despite BPTB autografts historically being the gold‐standard choice [22, 38, 62]. However, a meta‐analysis of 47,613 patients reported higher graft failure rates with HT versus BPTB autografts (OR = 0.83, *p* = 0.01), although the absolute difference was low (number needed to treat = 235 patients) [52]. This highlights the importance of individualizing graft selection according to patient preferences, as different individuals may weigh subjective and objective outcomes differently. Additionally, no included studies made reference to the increasingly popular quadriceps tendon (QT) autograft, which has demonstrated comparable outcomes to both HT and BPTB autografts [9, 58]. This paucity of data should be addressed in future studies to better guide surgical decision‐making. In terms of concomitant procedures, LETs have gained recent popularity as a method to prevent persistent rotational instability and relieve loading stress from the ACL graft, thus reducing the risk of re‐rupture [39, 54]. In fact, the recent STABILITY I trial demonstrated the addition of LETs during ACLR resulted in a statistically significant and clinically relevant reduction in graft failure and rotatory laxity [14]. Several other reviews have also endorsed the utility of LETs, and ongoing studies such as the STABILITY2 trial aim to validate their use with different graft choices [20, 43, 44]. This review is also in accordance with these findings and found that concomitant LETs increased the likelihood of achieving clinically meaningful outcomes by over 11‐fold. However, the study which reported this finding did not stratify by age group (i.e., pediatric vs. adult), sex, activity level or graft choice (cohort included BPTB autografts, BPTB allografts, HT autografts and QT autografts) [3]. This means that more research is required to fully characterize which patient characteristics and surgical factors enable LETs to truly confer benefit.

ML is a branch of artificial intelligence focused on self‐learning computer algorithms which create predictions in the absence of explicit instructions [10]. While traditional regression‐based models rely on assumptions such as linearity and additivity, ML models incorporate non‐linear relations and automatically modify effect estimates [15, 51]. Several studies have validated ML models for predicting clinically meaningful outcomes in orthopaedic surgery, specifically with total hip arthroplasty and hip arthroscopy [26, 28, 30]. This review included two studies which similarly support the ability of ML models to predict clinically meaningful outcomes following ACLR. The top‐performing models across the two studies demonstrated excellent predictive ability and identified similar predictive factors to the traditional regression models [29, 64]. Important future directions involve using ML and regression models for the development of a formal prognostic system for ACLR. Similar strategies have been used in the shoulder literature, including the ISIS and RoHI scores for anterior shoulder instability and rotator cuff repair, respectively [2, 32]. In order for this to be developed for the ACLR population, it is essential to identify what outcomes are relevant to defined ‘success’ (e.g., PROMs, re‐rupture and RTS) and is an ongoing topic of discussion via consensus groups [35, 57]. As ML models improve in the future, predictive capacity may also improve, leading to an improved understanding of the overall prognosis post‐ACLR.

This is the first review to assess the factors predicting achievement of the PASS, MCID or MIC for PROMs following ACLR. Regardless of the paucity of high‐quality evidence, summarizing the current literature regarding factors associated with clinically significant post‐ACLR outcomes provides a necessary framework for improved patient care. Limitations of this study mainly arose from the limited amount of evidence, with only 13 studies being included. Furthermore, the majority of included studies constituted Level III/IV evidence, with only one Level II study and no Level I studies. The lack of prospectively collected data also increases the risk of selection bias. There was also substantial heterogeneity with respect to the predictive variables, outcomes, and measures of clinical significance across the included studies. The relevance of the KOOS score in ACLR patients has also been questioned due to concerns of floor and ceiling effects, and lack of responsiveness [66]. This review also failed to explore the psychosocial aspect of ACL injuries. The traumatic injury and arduous recovery process can induce depressive symptoms, pain catastrophizing, kinesiophobia and similar issues, which have been shown to worsen post‐ACLR PROMs [63]. Moreover, the PROMs included in this review did not evaluate post‐operative mental health, which is a critical component of patient‐centred care [11]. Other limitations of this review included a lack of prospective registration, absence of quadriceps autograft data, absence of allograft data, and that patients were not stratified by age and activity level. Therefore, this review advocates for well‐designed prospective studies or RCTs to validate the predictors of clinically significant outcomes identified by this review and explore other potential predictors not presently addressed. These studies should aim to limit selection bias, employ varied statistical methods, ensure consistent thresholds of clinical significance, and include relevant evidence‐based outcome measures.

## CONCLUSION

Several factors predict the achievement of the PASS, MCID and MIC following ACLR. Higher pre‐operative PROMs, older age, male sex, lack of concomitant injuries, HT autograft use, concomitant LETs and higher post‐operative strength symmetry were the most important predictors of PASS achievement. Lower pre‐operative PROMs, male sex, non‐collision sports and lack of meniscectomies were the most common predictors of MCID or MIC achievement. This review provides a comprehensive understanding of the predictors of clinically significant post‐ACLR outcomes, thus providing a better framework for clinical decision‐making and managing patient expectations.

## AUTHOR CONTRIBUTIONS

Bryan Sun and Prushoth Vivekanantha conceived the study and contributed to study design, data acquisition, data analysis, data interpretation, manuscript drafting and manuscript revision. Hassaan Abdel Khalik and Darren de Sa critically revised the manuscript. All authors read and gave approval of the final manuscript to be published, and agree to be held accountable for all aspects of the work (i.e., ensuring the accuracy or integrity of any aspect of the work).

## FUNDING INFORMATION

The authors have no sources of funding to disclose.

## CONFLICT OF INTEREST STATEMENT

The authors declare no conflict of interest.

## ETHICS STATEMENT

There are no relevant ethical disclosures pertaining to research involving human participants and/or animals, and informed consent was not necessary to develop this manuscript.

## Supporting information

Supporting information.

## Data Availability

All data supporting the findings of this study are available within the paper and its Supplementary Information. The search strategy used to identify the included studies from online databases is shown in Supporting Information S1: Table 1.
