# Supplementary material for: Several factors predict the achievement of the patient acceptable symptom state and minimal clinically important difference for patient‐reported outcome measures following anterior cruciate ligament reconstruction: A systematic review
Source: Knee Surg Sports Traumatol Arthrosc. 2024 Sep 9;33(5):1617–32. doi: 10.1002/ksa.12460 (PMC12022816; doi:10.1002/ksa.12460)
Supplement: Supplementary file 1 — Supporting information. [file KSA-33-1617-s001.docx]

**SUPPLEMENTARY DIGITAL MATERIAL:**

**Supplementary Table 1.** Search Strategy

| 1. Anterior cruciate ligament injury/ or anterior cruciate ligament reconstruction/ or anterior cruciate ligament/ or ACL.mp or ACLR.mp |
| --- |
| 2. Hamstring tendon/ or hamstring muscle/ or hamstring*.mp |
| 3. Semitendinosus muscle/ or semitendinosus*.mp |
| 4. Minimal important change |
| 5. PASS |
| 6. Patient acceptable symptom state |
| 7. MID |
| 8. Minimal important difference |
| 9. ACL |
| 10. Anterior cruciate ligament |
| 11. 1 OR 2 OR 3 OR 4 OR 5 OR 6 OR 7 OR 8 |
| 12. 9 OR 10 |
| 13. 11 AND 12 |
